# The Impact of Family Socioeconomic Status on Preschoolers’ Anxiety: The Serial Mediation Effects of Parenting Style and Psychological Resilience in Preschoolers

**DOI:** 10.3390/bs15111443

**Published:** 2025-10-23

**Authors:** Limin Zhang, Yuxuan Xia, Siying Zhu, Xiaoxiao Lin, Jiaxin Xiang

**Affiliations:** 1 School of Education, Guangzhou University, Guangzhou 510006, China; zhanglimin333@126.com (L.Z.); 2112408138@e.gzhu.edu.cn (S.Z.); 2 Beicheng Kindergarten, Panyu District, Guangzhou 510006, China; 15602275830@163.com

**Keywords:** family socioeconomic status, parenting style, psychological resilience, preschooler

## Abstract

Anxiety is a common negative emotional experience among preschoolers that can significantly affect their physical and mental health development. Investigating the key factors that influence preschoolers’ anxiety and the mechanisms by which they act is important. This study aimed to examine the relationship between family socioeconomic status and preschoolers’ anxiety and explore the mediating role of parenting style and preschoolers’ psychological resilience in this relationship. This study examined the relationship between family socioeconomic status and childhood anxiety from the perspective of family factors and personal psychological characteristics. The Family Background Questionnaire, the Parenting Styles and Dimensions Questionnaire, the DECA-P2, and the Preschool Anxiety Scale were distributed to 36,048 parent–child dyads (children aged 3–6 years) in China. The collected data were analyzed via SPSS 22.0 and Mplus 8.3. A set of serial mediation models was constructed to provide evidence supporting the role of the key factors of early childhood anxiety and their observed associations. There were two-way correlations between family socioeconomic status (SES), parenting style, psychological resilience, and anxiety level. SES demonstrated a significant negative association with preschoolers’ anxiety, with direct and indirect links between authoritative and authoritarian parenting styles and preschoolers’ psychological resilience. Specifically, SES was associated with lower anxiety in preschoolers through authoritative parenting styles and was linked to higher anxiety through authoritarian parenting styles. SES was also related to preschoolers’ anxiety through their psychological resilience. In summary, parenting style and children’s psychological resilience function as serial mediators in the relationship between SES and preschoolers’ anxiety. Family socioeconomic status significantly and negatively correlates with early childhood anxiety, and parenting style and preschoolers’ psychological resilience mediate this relationship, in this study conducted from the perspectives of both family factors and individual psychological traits of preschoolers.

## 1. Introduction

The incidence of preschoolers’ psychological and behavioral problems and the prevalence of mental disorders have gradually increased. Anxiety disorders have emerged as a prevalent mental health challenge in early childhood, with profound implications for national public health systems and human capital development (Kwok et al., 2017). Among various anxiety disorders, generalized anxiety disorder (GAD) is particularly persistent, with symptoms often continuing into adolescence and adulthood (Johnston & Iarocci, 2017). GAD is a form of internalizing behavior problem in preschoolers and is one of the most common negative emotional experiences they encounter. It refers to the feelings of tension, fear, and worry that individuals experience when facing stressful situations (Noyes, 2002). These feelings are characterized primarily by physiological symptoms of tension and concerns about the future. Research indicates that anxiety is prevalent among preschool-aged children, with estimated prevalence rates ranging from 10 to 20% (Franz et al., 2013; Mian et al., 2012; Whalen et al., 2017).

Preschoolers’ anxiety not only significantly affects a young child’s current psychological adjustment but also predicts various psychological symptoms in adolescence and adulthood (LoParo et al., 2024; Steinsbekk et al., 2021). However, owing to preschoolers’ limited ability to verbally express discomfort or panic, their anxiety can often be misinterpreted (Whalen et al., 2017). If early anxiety is not promptly identified or addressed, it can lead to more severe psychological issues later in life (Pollard et al., 2023).

According to family systems theory, children develop through interactions and relationships with family members. Factors such as the family environment and parenting style significantly influence preschoolers’ emotional and behavioral development (Dougherty et al., 2013; Edwards et al., 2010; Zhang et al., 2022). Contemporary research adopts a multilevel perspective to examine childhood anxiety by integrating external environmental influences (e.g., family, school, and community) and intrinsic factors (e.g., children’s temperament) (Xiang et al., 2025; Degnan et al., 2010; Niditch & Varela, 2018). This ecological approach transcends single-factor analyses, instead of examining multiple perspectives within specific situations or across various contexts (Crnic et al., 2005). Although the above studies suggest that multiple family factors have a direct effect on anxiety in preschoolers, few studies have explored the mechanisms involved and have not considered the individual psychological characteristics of preschoolers. Thus, this study extends this framework by incorporating individual psychological resilience as a key mediator, thereby bridging macro-level family socioeconomic influences with micro-level child development processes, and explores the factors influencing preschoolers’ anxiety from two perspectives: family factors and individual psychological characteristics.

Family socioeconomic status (SES) is a critical variable within the family environment that profoundly affects preschoolers’ development and well-being (Bradley & Corwyn, 2002; Peverill et al., 2021; Zhu et al., 2019). SES measures parents’ social and economic standing in society and encompasses factors such as parental education level, income, and occupational prestige. Research has consistently shown that higher family SES is associated with better physical and mental health outcomes among preschoolers. For example, preschoolers from high-SES families tend to have greater access to healthcare, nutritious food, and educational resources, which collectively contribute to their overall well-being (Crnic et al., 2005). High-SES families often provide stimulating and supportive environments that promote cognitive and emotional development, thereby reducing anxiety risk. Conversely, preschoolers with lower-SES backgrounds are more likely to exhibit behavioral problems and frequently encounter chronic stressors and long-term stress regulatory burdens (Strickhouser & Sutin, 2020). These stressors include financial instability, poor housing conditions, and limited access to healthcare and educational opportunities. Such environments can lead to increased exposure to adverse experiences and a greater likelihood of developing negative emotions, such as anxiety (Luo et al., 2023; Roubinov et al., 2018). Chronic stress associated with low-SES environments can impair the development of stress-regulatory mechanisms in preschoolers, increasing their vulnerability to anxiety and other mental health issues (Evans & Kim, 2007). Thus, family SES may influence preschoolers’ anxiety through specific mechanisms related to access to resources and exposure to chronic stress. On the basis of this understanding, we propose

**Hypothesis** **1:** *Family SES significantly predicts anxiety levels in preschoolers*.

Differences in family SES influence parents’ values and expectations for themselves and their children, leading to variations in parenting styles. Parenting style refers to the behavioral tendencies of parents in raising their children and is characterized by relative stability and consistency across different contexts. It encompasses the attitudes and behaviors that parents exhibit toward their children (Darling & Steinberg, 1993). According to Baumrind (1967), parenting styles can be categorized as authoritative, authoritarian, or permissive based on the nature of parent–child interactions. Research indicates a correlation between parenting style and anxiety levels among preschoolers. Authoritative parenting was significantly negatively associated with preschoolers’ anxiety, whereas authoritarian parenting was significantly positively correlated with anxiety (Delvecchio et al., 2020; Heaton et al., 2023). Parents with a high SES mostly adopt a positive authoritative parenting style for their young children (Niditch & Varela, 2018), which allows preschoolers to have more stable emotional control and to show more confidence and sympathy (Baumrind, 2012). In contrast, parents with a low SES are more likely to adopt a harsh or punitive authoritarian parenting style (Roubinov & Boyce, 2017), which often deprives parents of emotional communication with their preschoolers, resulting in their vulnerability to anxiety and fear (Cano, 2022). Based on this understanding, we propose

**Hypothesis** **2:** *Parenting style plays a mediating role in the effect of family socioeconomic status on preschoolers’ anxiety.*

In addition, the influence of the external environment on preschoolers’ development largely depends on their own characteristics (Gómez et al., 2024). With the rise of positive psychology research that has turned to the study of human potential and capabilities, psychological resilience, one of the psychological attributes of individuals, has received researchers’ attention. Psychological resilience has been translated into mental elasticity, resilience, and resistance (Masten & Coatsworth, 1998). The process of action is a dynamic mechanism involving interactions between internal and external protection and risk processes (Rutter, 1999). This action results in a phenomenon that enables an individual to experience adversity and challenges without being physically and mentally adversely affected and successfully adapting (Masten, 2001; Werner, 1993). Although some individuals face severe stress or adversity in childhood, they still grow up unaffected or even go on to lead a flourishing life (Ho et al., 2024). Thus, the negative effects of family factors on preschoolers may be altered by the effects of psychological resilience (Hao et al., 2015; Howell et al., 2020; Poole et al., 2017; Sheerin et al., 2018). In other words, psychological resilience mitigates anxiety in preschoolers. In models of preschoolers’ psychological resilience development, poverty, social support, and parenting involvement are important predictors of their perceived self-efficacy, self-efficacy in relation to others, etc., which results in either negative (antisocial behaviors and depression) or positive (pro-social behaviors) performance (Y. Wang et al., 2024). A high level of parental education is a protective factor for the development of preschoolers’ psychological resilience (Waanders et al., 2007), whereas factors such as family poverty and low levels of parental education can result in a negative development of psychological resilience, which can lead to the development of serious psychological, behavioral, and social problems in adolescents (Çelik, 2024; Tolan & Ugur, 2024). Therefore, this study proposes

**Hypothesis** **3:** *Psychological resilience mediates the effect of family socioeconomic status on preschoolers’ anxiety.*

Moreover, parenting style significantly influences preschoolers’ mental toughness (Hoffman, 2010). Positive parenting styles are the most important protective factor for preschoolers’ psychological resilience (Li et al., 2019), and parenting that tends to be authoritative and positive helps children develop psychological resilience and cope effectively with stress (J. Xu et al., 2024). Authoritarian parenting, such as harsh punishment and refusal of commands, reduces preschoolers’ psychological resilience scores (Morgan et al., 2020). A study of older adults in China revealed that older adults whose parents favored positive and authoritative parenting styles had higher levels of psychological resilience and lower levels of depression and anxiety, whereas older adults who were raised in an authoritarian parenting style had higher levels of depression and anxiety and lower levels of psychological resilience (Ding et al., 2023). Parenting styles are the primary resource for promoting the development of psychological resilience in preschoolers, thus providing a pristine environment for development (Chari & Chandrashekhar, 2014). These studies suggest that parenting style may affect preschoolers’ anxiety through the mediating effect of psychological resilience. Therefore, this study proposes Hypothesis 4: early childhood psychological resilience mediates the relationship between parenting styles and preschoolers’ anxiety.

**Figure 1:**
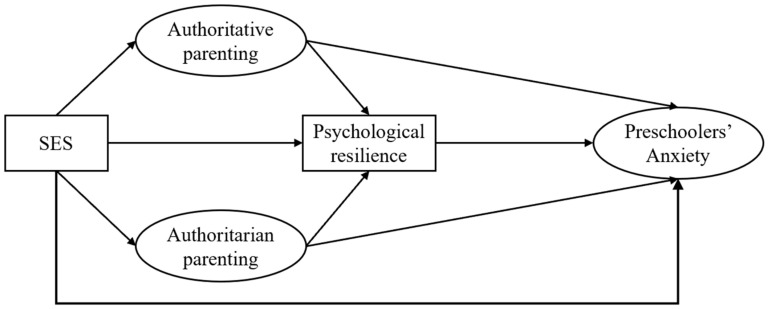
Hypothesized model of family socioeconomic status influencing anxiety in preschoolers.

While prior research has established direct links between socioeconomic status (SES) and childhood anxiety and has separately identified the roles of parenting styles and psychological resilience, little research has systematically examined the sequential mediating pathways through which these factors may jointly mitigate the effects of socioeconomic disparities on preschoolers’ anxiety (Xiang et al., 2025; Lin et al., 2024). Furthermore, existing research has focused mostly on school-aged children, neglecting preschoolers (He et al., 2025; Liu et al., 2024; X. Xu et al., 2025). Therefore, this study proposes a multiple mediation model in which parenting styles and preschoolers’ psychological resilience act as chain mediators between family socioeconomic status and preschoolers’ anxiety. The exploration of this issue contributes to a deeper understanding of the relationship between family socioeconomic status and preschoolers’ anxiety. The hypothesized model is illustrated in Figure 1.

## 2. Materials and Methods

### 2.1. Participants

A cross-sectional survey-based study was conducted online. Parents from Guangdong, Hubei, Henan, and Guangxi Provinces in China were included in this study. A convenience sampling technique was employed due to the survey’s online nature, with the sample size determined by the availability of respondents during the data collection period. A total of 36,793 responses were collected, of which 36,048 were valid, resulting in a high response rate of 97.98%. All abnormal data were cleaned, including (1) responses to single questions completed in less than 2 s, and (2) children outside the 3–6 age range. Of these, 78.9% were reported by mothers. Among the preschoolers, 53.7% were boys, and 72.3% were under the age of 5. The total income of these families fell mainly into the categories of RMB 30,000–80,000 and RMB 80,000–150,000 (accounting for 24.1% and 25.6%, respectively). In terms of educational background, a relatively even distribution was observed among parents with qualifications ranging from junior high school (accounting for 21.8% of mothers and 20.2% of fathers) to undergraduate (accounting for 24.9% of mothers and 27.1% of fathers), whereas only a small percentage received education in primary school and below (accounting for 2.6% of mothers and 1.9% of fathers) or postgraduate and above (accounting for 3.1% of mothers and 4.4% of fathers). The majority of the surveyed parents held a bachelor’s degree. For job distribution among parents, a similar pattern was observed for fathers and mothers, 44.5% and 45% of whom worked as general managers or professional technicians, respectively, accounting for the largest share of the types of jobs presented. The participants’ demographic information is presented in Table 1.

### 2.2. Measures

#### 2.2.1. Family’s Socioeconomic Status (SES)

This study measured each family’s socioeconomic status (SES) by assessing the total income, education, and occupation of all family members. Specifically, families with total incomes ranging from RMB 30,000 to RMB 250,000 and above were assigned scores of 1 to 5, respectively. Similarly, a 6-point scale was used for both educational background and occupation. For educational background, scores ranged from primary school and below to postgraduate and above, while for occupation, scores ranged from temporary or unemployed workers to senior managers and professional technicians.

Drawing on PISA’s calculation method for the SES of the students’ families, the average values of parents’ careers and education were taken as indicators of family occupation and educational background, respectively, and were calculated with the total family income for the standard scores. Principal component analysis was used to extract three factors, and the characteristic value of the largest factor was 2.13. The factor loads for family education, occupation, and total annual family income were 0.84, 0.84, and 0.84, respectively. The standard scores of the three indicators were weighted by factor loading and summed. Finally, the preschoolers’ family characteristics were divided by the factor’s characteristic value.

#### 2.2.2. Parenting Styles

The Parenting Styles and Dimensions Questionnaire (PSDQ), developed by Robinson et al. (1995), with the Chinese version revised by Cheah et al. (2009), was used in this study to assess parenting styles. The original questionnaire includes three subscales: authoritative, authoritarian, and permissive parenting. Each question is scored on a 5-point Likert scale ranging from ‘never’ to ‘always’ (1–5 points). Higher scores indicate a stronger tendency toward parenting styles. Studies have shown that permissive parenting is less common in China (Wu et al., 2002); therefore, only authoritative and authoritarian parenting styles were considered in this study.

The authoritative subscale contains 4 dimensions: warm acceptance (11 questions), understanding and guidance (7 questions), encouragement and democracy (5 questions), and warm response (4 questions) (e.g., “I encourage my child to talk about his/her troubles.”). In this study, the overall Cronbach’s α coefficient for the authoritative parenting scale was 0.922, with α coefficients of 0.83, 0.80, 0.69, and 0.66.

The authoritarian subscale includes four dimensions: malicious words (four questions), corporal punishment (six questions), arbitrary punishment (six questions), and commands (four questions) (e.g., I yell at my child when he/she misbehaves.”). Question 30, ‘Tells child what to do,’ was removed because of a negative correlation. After its removal, the Cronbach’s alpha coefficient increased from 0.60 to 0.73. Finally, 19 questions remained, and the Cronbach’s α of the total scale of authoritarian education was 0.90, with 0.62, 0.79, 0.79, and 0.73 as the corresponding coefficients for each dimension.

#### 2.2.3. Preschoolers’ Psychological Resilience

Preschoolers’ psychological resilience was assessed via the Devereux Scale (DECA-P2) developed by LeBuffe and Naglieri (2013) and the Chinese version revised by Ji et al. (2015). The original scale contains 38 items, which are divided into four dimensions: initiative (9 questions), self-regulation (9 questions), attachment/relationships (9 questions), and behavioral problems (11 questions). The first three dimensions measure preschoolers’ psychological resilience, whereas the fourth assesses their implicit or explicit problematic behaviors. Therefore, this study focused mainly on the first three dimensions (e.g., “Keep trying when something doesn’t work out.”).

Parents rate their child’s behaviors on a scale from ‘1—never’ to ‘5—always,’ based on the frequency of their corresponding behaviors. Higher scores indicate better psychological resilience. The scoring method followed the T-score conversion method recommended by LeBuffe and Naglieri (2013), which converts the original scores of initiative, self-regulation, and attachment/relationships into T-scores. T-scores were then summed to obtain the overall protective factor subscale scores. The formula used for T-score conversion was T = 50 + 10z, where z represents the standard score. The z-values in this study were calculated based on the sample parameters, whereas the original scale used foreign constant-model parameters.

#### 2.2.4. Preschoolers’ Anxiety

Preschoolers’ anxiety was measured via the Preschool Anxiety Scale (PAS) developed by Spence et al. (2001), with the Chinese version revised by M. Wang et al. (2009). The original scale consists of 28 items divided into five dimensions: generalized anxiety, social anxiety, obsessive–compulsive disorder, physical injury fears, and separation anxiety. Because of the focus on generalized anxiety, only the ‘generalized anxiety’ dimensions (5 items) from this dimension out of a total of 28 items in all 5 dimensions were used in this study (e.g., “He/She finds it hard to stop worrying/being upset.”). The participants were scored on a five-point scale ranging from (‘never’) to (‘always’), with higher scores indicating more severe symptoms of anxiety. The alpha and omega coefficients were 0.87 and 0.87, respectively.

### 2.3. Procedure and Data Processing

In cooperation with the China regional departments of education, this study distributed online questionnaires to parents at affiliated kindergartens, asking them for voluntary participation in this study, and obtained informed consent from them. The guidance section of the questionnaire explained the survey’s purpose and the implementing unit and informed parents that feedback on their parenting and children’s development would be provided upon survey completion. This approach encouraged parents to complete the questionnaire as accurately as possible. This study involving human subjects was approved by Guangzhou University’s School of Education Research Ethics Committee, and common ethical practices in research with human subjects were followed.

Analyses were conducted via SPSS 25.0 and Mplus 8.3 in several steps: (1) Harman’s single-factor analysis was used to test the common method deviation; (2) descriptive statistics and correlations among variables were analyzed; (3) the main effects between SES, preschoolers’ anxiety, and control variables were analyzed; and (4) the mediating effects of preschoolers’ parenting style and psychological resilience were analyzed.

## 3. Results

### 3.1. Common Method Deviation Test

Since the data analysis was in the form of parents’ reports, there may have been a potential common method bias effect. Therefore, Harman’s single-factor analysis was used to test for common method bias. Eleven principal components were extracted before factor rotation using principal component analysis. The first factor explained 21.974% of the total variance, which was less than the critical value of 40%, indicating that there was no serious common method deviation in the data analysis (Podsakoff et al., 2003).

### 3.2. Descriptive Statistics and Correlation Analysis

Table 2 shows the correlation matrix between the variables based on the observation scores. Preschoolers’ anxiety was significantly negatively correlated with family SES (*β* = −0.06; *p* < 0.001), parents’ authoritative parenting (*β* = −0.19; *p* < 0.001), and preschoolers’ psychological resilience (*β* = −0.16; *p* < 0.001), but was positively correlated with parents’ authoritarian parenting (*β* = 0.31; *p* < 0.001). Conversely, preschoolers’ psychological resilience was positively correlated with their family SES (*β* = 0.23; *p* < 0.001) and authoritative parenting (*β* = 0.53; *p* < 0.001). However, it was negatively correlated with authoritarian parenting (*β* = −0.30; *p* < 0.001).

In general, there were pairwise correlations between family SES, authoritative and authoritarian parenting, preschoolers’ psychological resilience, and preschoolers’ anxiety levels, which provided the necessary information for the subsequent analysis.

### 3.3. Main Effect and Mediating Effects

First, to verify the main effect between preschoolers’ anxiety and the dependent variables, a regression model with SES, preschoolers’ anxiety, and control variables (i.e., preschoolers’ age and gender) revealed that the main effect of SES on preschoolers’ anxiety was −0.03 (*p* < 0.001).

**Figure 2:**
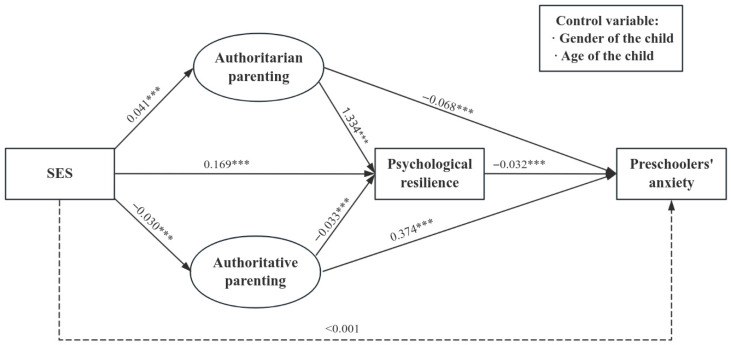
Intermediary model diagram (note: *** *p* < 0.001).

Second, Figure 2 shows the intermediary effect tests for all the samples, and the coefficients on the path are standardized regression coefficients. The results show that there was a good degree of fit between the model and the statistics, with (106) = 17,167.54, CFI = 0.94, TLI = 0.93, RMSEA = 0.07, 90% CI = [0.07, 0.07], and SRMR = 0.04. After controlling for preschoolers’ age and gender, SES significantly predicted changes in authoritative parenting (*β* = 0.04; *p* < 0.001), authoritarian parenting (*β* = −0.03; *p* < 0.001), and psychological resilience (*β* = −0.17; *p* < 0.001). Authoritative parenting (*β* = 1.33; *p* < 0.001) and authoritarian parenting (*β* = −0.13; *p* < 0.001) significantly predicted changes in preschoolers’ psychological resilience. Authoritative parenting (*β* = −0.07; *p* < 0.001), authoritarian parenting (*β* = 0.37; *p* < 0.001), and preschoolers’ psychological resilience *(β* = −0.03; *p* < 0.001) strongly affected their anxiety levels. However, the direct effect of SES on preschoolers’ anxiety levels was not statistically significant (*β* < 0.001; *p* = 0.95).

Five thousand rounds of repeated sampling using the bootstrap method were conducted to test the intermediary effects of the two parenting styles and psychological resilience on SES and anxiety levels in preschoolers. The results are presented in Table 3, showing that the indirect effect of SES on preschoolers’ anxiety through authoritative parenting was −0.003 (95% CI = −0.004; −0.002). The indirect effect of SES on preschoolers’ anxiety through authoritarian parenting was −0.011 (95% CI = −0.014; −0.009), whereas that through psychological resilience was −0.005 (95% CI = −0.007; −0.004). Furthermore, the indirect effects of SES on preschoolers’ anxiety through authoritative or authoritarian parenting and then through psychological resilience were −0.002 (95% CI = −0.002; −0.001) and <0.001 (95% CI = 0.000; 0.000), respectively. The confidence intervals of the five intermediary effects did not contain zero, indicating a significant intermediary effect of authoritative parenting, authoritarian parenting, and preschoolers’ psychological resilience on SES and preschoolers’ anxiety. SES can affect preschoolers’ anxiety through any of the three factors, namely, authoritative parenting, authoritarian parenting, and psychological resilience. It can also influence preschoolers’ anxiety by affecting either of the two parenting styles first, and then through psychological resilience to preschoolers’ anxiety.

## 4. Discussion

### 4.1. Mediation of Parenting Style on Preschoolers’ Anxiety

The results show that family SES can be significantly associated with preschoolers’ anxiety through the mediation of authoritative and authoritarian parenting. Family SES was found to be negatively associated with preschoolers’ anxiety through the mediation of authoritative parenting, but it showed positive associations with preschoolers’ anxiety through the mediation of authoritarian parenting, which is consistent with existing research results (Heaton et al., 2023). The educational level or professional factors related to parents’ SES are associated with their parenting style (Baumrind, 2012). Parents with a high SES tend to adopt authoritative parenting, which is represented by accepting preschoolers’ opinions carefully, giving them understanding and emotional warmth, and striking a balance between giving preschoolers freedom and education (Baumrind, 1971), which is likely to promote their children’s well-being (Podsakoff et al., 2003).

In contrast, a low SES is associated with disruptions in parents’ well-being and parenting quality, and parents are likely to adopt an authoritarian parenting style, which is characterized by being too strict, insensitive to preschoolers’ needs and opinions, and highly manipulative. They expect children to follow their specific and strict rules and often punish them (Hutchison et al., 2016), which is likely to place children at heightened risk for developmental difficulties, including anxiety, conduct problems, and poor socioemotional competence (Niditch & Varela, 2018).

### 4.2. Mediation of Preschoolers’ Psychological Resilience on Preschoolers’ Anxiety

The results show that family SES was associated with preschoolers’ anxiety through their psychological resilience. The positive impact of family SES on preschoolers’ psychological resilience is consistent with the findings of previous research (Masten et al., 1988). The higher the SES of the family, the better the preschoolers’ psychological resilience. This finding supports ecosystem theory, which states that external environmental variables need to work through individual-level variables and that the impact on preschoolers varies with the development of individual characteristics (Ashiabi & O’Neal, 2015). The dynamic model of psychological resilience provides a good explanation. Socioeconomic advantages and parents’ education above secondary school promote and protect the development of preschoolers’ psychological resilience, whereas poverty serves as a hazardous factor in this course (M. O. Wright & Masten, 2005). In self-determination theory, social support is recognized as one of the key factors in meeting an individual’s basic psychological needs, and when individuals feel support from others, they are more likely to experience a sense of worth and security, which helps them cope with adversity more effectively (Ryan & Deci, 2000); that is, support from others improves the level of psychological resilience, which enables preschoolers to feel intimate relationships between themselves and others, thus alleviating negative emotions (Rutter, 1999). When families are economically advantaged in an external environment, anxiety can be reduced by promoting the development of preschoolers’ psychological resilience, whereas when families are economically disadvantaged, anxiety can be reduced through preschoolers’ psychological resilience to effectively cope with difficult challenges.

### 4.3. Chain Mediation Between Parenting Style and Preschooler Psychological Resilience on Preschoolers’ Anxiety

The results show that family SES was associated with preschoolers’ anxiety via the chain-mediating effect of parenting style and preschoolers’ psychological resilience. This multiple-mediating effect indicates that external environmental and internal individual factors follow a specific sequential pattern in their relationship with preschool anxiety. Moreover, preschoolers’ psychological resilience serves as an intermediary variable between parenting style and anxiety, which is consistent with existing research (Chorpita & Barlow, 1998). These conclusions support family systems theory, which considers human emotions and communication behavior and regards the whole family as an emotional unit in which the activities of family members are interactive. Within this pattern, parents’ interactive parenting style is directly linked to preschoolers’ psychological functions and, consequently, correlates with their emotional performance (Muhtadie et al., 2013).

Family SES was associated with preschoolers’ anxiety via the authoritative parenting style and preschoolers’ psychological resilience. This finding verified the protective and promoting effects of authoritative parenting on psychological resilience. The dynamic model of psychological resilience indicates that preschoolers’ psychological resilience can be developed only when their psychological needs for safety, love, belonging, value, and control are satisfied (Evans & Kim, 2007). Authoritative parenting refers to a strong sense of care, a good parent–child relationship, and moderate expectations, which are protective factors that can meet the psychological needs of preschoolers and facilitate psychological resilience (Steinberg et al., 1994). Research also shows that the more protective factors preschoolers obtain from their families, the stronger their psychological resilience, and the more they can withstand setbacks and stress (Bandura et al., 2001). Parents’ authoritative parenting style meets the needs of children’s love, belonging, and sense of control, such as ‘I will encourage children to talk about the difficulties encountered so that they can have a sense of security and enhance their psychological resilience (Chao, 1994). Psychological resilience is a dynamic process that is constantly adapted and adjusted to changes in the environment, helping individuals face adversity positively. Therefore, even in low-SES contexts, preschoolers’ psychological resilience can improve via parental authority, which may correspond to reduced anxiety levels.

Conversely, family SES was associated with preschoolers’ anxiety via parents’ authoritarian parenting styles and preschoolers’ psychological resilience. Preschoolers’ psychological resilience is a mediator between parents’ authoritarian parenting style and preschoolers’ anxiety, which is consistent with existing research conclusions (M. O. Wright & Masten, 2005). Authoritarian parenting is a risk factor for preschoolers’ psychological resilience, as parent–child attachment is usually poor and cannot meet the basic psychological needs of children (Pinquart, 2017). Behaviors such as ‘I will be angry with children’ and ‘I do not agree with children’s ideas’ reflect the autocratic adult perspective of the parents, which adversely affects children’s self-confidence and leads to anxiety. However, the mediating effect of psychological resilience alleviates the anxiety caused by authoritarian parenting (Williams et al., 2012). Bandura’s social cognitive theory proposes that the subjective factors of individuals are important elements that determine one’s behavioral tendencies rather than passively depending on the external environment (Bandura, 1989). Although low SES and authoritarian parenting styles affect children’s emotions, their psychological resilience can reduce anxiety, which, to some extent, indicates that children can recover from adversity. Masten and Coatsworth (1998) suggested that the study of the effects of psychological resilience on children’s positive development can help eliminate some fatalism and that a child’s future personality, capacity, and prospects should not be determined in infancy. Just as attention is given to the value of human beings in humanistic psychology, people are born with a motivation for growth and development. Given a good psychological environment, such as warmth, sympathy, and support, everyone will find their true self and give full play to their creativity and potential (Rutter, 1999).

### 4.4. Implications

This study explored the influence of family factors and preschoolers’ psychological characteristics on preschoolers’ anxiety, providing a reference for improving family education. Education departments and relevant preschool education institutions should incorporate family education guidance services into their work plans and teacher training, and provide parents with targeted family education programs. In addition, government departments should provide employment assistance to parents with a low socioeconomic status to create the conditions for them to implement family education. Simultaneously, government departments and kindergartens should set up parental guidance schools and provide them with public welfare and professional family education guidance services via internet platforms to guide parents in changing their parenting style. Furthermore, research tools, such as scientific and systematic scales or observations, should be used to monitor the psychological development of preschoolers and pay attention to their physical and mental health.

### 4.5. Limitations and Future Research

Several limitations remain in this study. First, treating the family as a unit might cover the differences between different family members in terms of family SES and parenting style. Second, the empirical data of this study were obtained from behavioral indicators, whereas the development of psychological resilience emphasized ecological principles. The mechanism of psychological resilience calls for an interplay between physiology and psychology, whose influencing factors should also be guided by the concept of system development, encompassing an ecosystem of individual, family, social networks, and other interactions (Kumpfer, 2002). Theories and mechanistic models of psychological resilience established without considering biological factors are not comprehensive (Mahoney et al., 2014). For example, MRI, EEG, and other relevant technologies can be used to provide more comprehensive information. Third, this study was a cross-sectional study of parental self-reports and thus could not adequately account for the causal relationships between variables. Subsequent longitudinal studies will be needed to shed light on causal relationships.

Future research could address the effects of cultural context on preschoolers’ anxiety to explore other potential predictors. For example, the effects of the parenting styles of different family members, such as fathers or mothers, on preschoolers’ anxiety could be examined. The effects of family SES on parenting style and preschoolers’ psychological resilience in different cultural contexts could be examined to verify the universality and specificity of these relationships. Additionally, more attention should be given to the measurement of physiological indicators in the course of research. A key issue is that previous research has shown that parental mental health problems, including childhood anxiety disorders, have a significant effect on the development of internalizing symptoms (Peng et al., 2022; K. Wright et al., 2017). To address this issue, future research could test the potential impact of parental mental health issues by using the current research model. Additionally, future research should consider a longitudinal study design to observe the changes in these variables across developmental stages and their long-term effects on preschoolers’ anxiety levels. Finally, an important practical direction for researchers is to develop interventions tailored to different family backgrounds to help parents adopt more effective parenting styles, thereby promoting the mental health and resilience development of preschoolers.

## 5. Conclusions

This study demonstrated that family socioeconomic status is significantly and negatively associated with early childhood anxiety, with mediating associations between parenting styles and psychological resilience from the perspectives of both family factors and the individual psychological traits of preschoolers. Furthermore, this study constructed a set of action chain models to provide evidence supporting the study of the key factors of early childhood anxiety and their action mechanisms. Our findings highlight the important role of psychological resilience in buffering the impact of socioeconomic stress on preschoolers’ mental health, providing empirical support for the application of psychological resilience theory in the field of early childhood development. The results extend the current frameworks of family parenting theories and the stress attenuation model by demonstrating interactive associations between parenting styles and psychological resilience across socioeconomic contexts. These relationships have implications for the development of prevention and intervention strategies targeting childhood anxiety.

## Figures and Tables

**Table 1:** Distribution of survey sample in terms of demographic variables (*n* = 36,048).

Variable	*n* (%)	Variable	*n* (%)	
Sex		Parental education	Father	Mother
Boy	19,375 (53.7)	Primary school and below	681 (1.9)	945 (2.6)
Girl	16,673 (46.3)	Junior high school	7274 (20.2)	7848 (21.8)
Age		High school and technical secondary school	8630 (23.9)	8612 (23.9)
3 years old	3910 (10.8)	Junior college	8105 (22.5)	8566 (23.8)
4 years old	10,497 (29.1)	Undergraduate	9768 (27.1)	8976 (24.9)
5 years old	11,676 (32.4)	Postgraduate and above	1590 (4.4)	1101 (3.1)
6 years old	9965 (27.6)	Parental occupation		
Reported by		Temporary workers and non-engaged workers	2076 (5.8)	7452 (20.7)
Father	7621 (21.1)	Unskilled workers, and agricultural and forestry laborers	1607 (4.5)	1858 (5.2)
Mother	28,427 (78.9)	Manual laborers, skilled workers, and service workers	9231 (25.6)	7317 (20.3)
Total household income		General managers and professional technicians	16,035 (44.5)	16,226 (45.0)
30,000 and below	4729 (13.1)	Middle managers	5006 (13.9)	2606 (7.2)
30,000–80,000	8676 (24.1)	Senior managers	2093 (5.8)	589 (1.6)
80,000–150,000	9216 (25.6)			
150,000–250,000	6420 (17.8)			
Above 250,000	7007 (19.4)			

**Table 2:** Descriptive statistics and correlation matrix of core variables (*n* = 36,048).

	1.	2.	3.	4.	5.	6.	7.
1. SES	1						
2. Authoritative parenting	0.10 ***	1					
3. Authoritarian parenting	−0.05 ***	−0.42 ***	1				
4. Psychological resilience	0.23 ***	0.53 ***	−0.30 ***	1			
5. Preschoolers’ anxiety	−0.06 ***	−0.19 ***	0.31 ***	−0.16 ***	1		
6. Gender	0.01 *	0.03 ***	−0.06 ***	0.07 ***	0.00	1	
7. Age	−0.16 ***	−0.04 ***	0.05 ***	−0.04 ***	0.08 ***	−0.02 ***	1
Mean	0	4.15	2.31	150	1.81	-	-
Standard deviation	1	0.44	0.65	26.86	0.67	-	-

* means *p* < 0.05, *** means *p* < 0.001.

**Table 3:** Mediating effects of parenting style and psychological resilience.

Mediated Pathways	Estimates of Indirect Effect	95% Confidence Interval	
		Lower	Upper
SES → authoritative parenting → anxiety	−0.003 (0.001)	−0.004	−0.002
SES → authoritarian parenting→ anxiety	−0.011 (0.001)	−0.014	−0.009
SES → psychological resilience → anxiety	−0.005 (0.001)	−0.007	−0.004
SES → authoritative parenting → psychological resilience → anxiety	−0.002 (<0.001)	−0.002	−0.001
SES → authoritarian parenting → psychological resilience → anxiety	<0.001 (<0.001)	<0.001	<0.001

## Data Availability

The data supporting the findings of this study are available upon request from the corresponding author.

## Abbreviations

The following abbreviations are used in this manuscript:

SES	socioeconomic status
GAD	generalized anxiety disorder
DECA-P2	Devereux Scale

## References

[B1-behavsci-15-01443] Ashiabi G. S., O’Neal K. K. (2015). Child social development in context: An examination of some propositions in Bronfenbrenner’s bioecological theory. Sage Open.

[B2-behavsci-15-01443] Bandura A. (1989). Human agency in social cognitive theory. The American Psychologist.

[B3-behavsci-15-01443] Bandura A., Barbaranelli C., Caprara G. V., Pastorelli C. (2001). Self-efficacy beliefs as shapers of children’s aspirations and career trajectories. Child Development.

[B4-behavsci-15-01443] Baumrind D. (1967). Child care practices anteceding three patterns of preschool behavior. Genetic Psychology Monographs.

[B5-behavsci-15-01443] Baumrind D. (1971). Current patterns of parental authority. Developmental Psychology.

[B6-behavsci-15-01443] Baumrind D. (2012). Differentiating between confrontive and coercive kinds of parental power-assertive disciplinary practices. Human Development.

[B7-behavsci-15-01443] Bradley R. H., Corwyn R. F. (2002). Socioeconomic status and child development. Annual Review of Psychology.

[B8-behavsci-15-01443] Cano T. (2022). Social class, parenting, and child development: A multidimensional approach. Research in Social Stratification and Mobility.

[B9-behavsci-15-01443] Chao R. K. (1994). Beyond parental control and authoritarian parenting style: Understanding Chinese parenting through the cultural notion of training. Child Development.

[B10-behavsci-15-01443] Chari U., Chandrashekhar P. (2014). Parents: A resource to promote resilience in children. Asian Journal of Psychiatry.

[B11-behavsci-15-01443] Cheah C. S., Leung C. Y., Tahseen M., Schultz D. (2009). Authoritative parenting among immigrant Chinese mothers of preschoolers. Journal of Family Psychology.

[B12-behavsci-15-01443] Chorpita B. F., Barlow D. H. (1998). The development of anxiety: The role of control in the early environment. Psychological Bulletin.

[B13-behavsci-15-01443] Crnic K. A., Gaze C., Hoffman C. (2005). Cumulative parenting stress across the preschool period: Relations to maternal parenting and child behaviour at age 5. Infant and Child Development.

[B14-behavsci-15-01443] Çelik A. (2024). Current research trends in child poverty and psychological resilience research: A bibliometric analysis approach. Sustainable Development.

[B15-behavsci-15-01443] Darling N., Steinberg L. (1993). Parenting style as context: An integrative model. Psychological Bulletin.

[B16-behavsci-15-01443] Degnan K. A., Almas A. N., Fox N. A. (2010). Temperament and the environment in the etiology of childhood anxiety. Journal of Child Psychology and Psychiatry.

[B17-behavsci-15-01443] Delvecchio E., Germani A., Raspa V., Lis A., Mazzeschi C. (2020). Parenting styles and child’s well-being: The mediating role of the perceived parental stress. Europe’s Journal of Psychology.

[B18-behavsci-15-01443] Ding X., Zheng L., Liu Y., Zhang W., Wang N., Duan H., Wu J. (2023). Parenting styles and psychological resilience: The mediating role of error monitoring. Biological Psychology.

[B19-behavsci-15-01443] Dougherty L. R., Tolep M. R., Bufferd S. J., Olino T. M., Dyson M., Traditi J., Rose S., Carlson G. A., Klein D. N. (2013). Preschool anxiety disorders: Comprehensive assessment of clinical, demographic, temperamental, familial, and life stress correlates. Journal of Clinical Child & Adolescent Psychology.

[B20-behavsci-15-01443] Edwards S. L., Rapee R. M., Kennedy S. (2010). Prediction of anxiety symptoms in preschool-aged children: Examination of maternal and paternal perspectives. Journal of Child Psychology and Psychiatry.

[B21-behavsci-15-01443] Evans G. W., Kim P. (2007). Childhood poverty and health: Cumulative risk exposure and stress dysregulation. Psychological Science.

[B22-behavsci-15-01443] Franz L., Angold A., Copeland W., Costello E. J., Towe-Goodman N., Egger H. (2013). Preschool anxiety disorders in pediatric primary care: Prevalence and comorbidity. Journal of the American Academy of Child & Adolescent Psychiatry.

[B23-behavsci-15-01443] Gómez G., Rivas M., Giaconi V., Martínez C., Burrone M. (2024). Understanding the influence of children’s mental health, cognitive development, and environmental factors on learning outcomes in Chile. Humanities & Social Sciences Communications.

[B24-behavsci-15-01443] Hao S., Hong W., Xu H., Zhou L., Xie Z. (2015). Relationship between resilience, stress and burnout among civil servants in Beijing, China: Mediating and moderating effect analysis. Personality and Individual Differences.

[B25-behavsci-15-01443] He Y., Hu J., Shen Y., Yu T. (2025). Family socioeconomic status and psychological capital among Chinese children: Roles of parental burnout and family function. Journal of Child and Family Studies.

[B26-behavsci-15-01443] Heaton K. G., Camacho N. L., Gaffrey M. S. (2023). Associations between pre-pandemic authoritative parenting, pandemic stressors, and children’s depression and anxiety at the initial stage of the COVID-19 pandemic. Scientific Reports.

[B27-behavsci-15-01443] Ho G., Leung D., Chan A., Bressington D., Karatzias T. (2024). How do you become resilient? A critical realist explanation of the youth resilience process. Adversity and Resilience Science.

[B28-behavsci-15-01443] Hoffman D. (2010). Risky investments: Parenting and the production of the “resilient child”. Health Risk & Society.

[B29-behavsci-15-01443] Howell K., Miller-Graff L., Schaefer L., Scrafford K. (2020). Relational resilience as a potential mediator between adverse childhood experiences and prenatal depression. Journal of Health Psychology.

[B30-behavsci-15-01443] Hutchison L., Feder M., Abar B., Winsler A. (2016). Relations between parenting stress, parenting style, and child executive functioning for children with ADHD or autism. Journal of Child & Family Studies.

[B31-behavsci-15-01443] Ji Y., Niu Y., Tang Z., Yang H. (2015). Validity and reliability of the Chinese version of the Devereux early childhood assessment for preschoolers second edition. Chinese Mental Health Journal.

[B32-behavsci-15-01443] Johnston K., Iarocci G. (2017). Are generalized anxiety and depression symptoms associated with social competence in children with and without autism spectrum disorder?. Journal of Autism and Developmental Disorders.

[B33-behavsci-15-01443] Kumpfer K. L. (2002). Factors and processes contributing to resilience.

[B34-behavsci-15-01443] Kwok S., Gu M., Cheung A. (2017). A longitudinal study of the role of children’s altruism and forgiveness in the relation between parental aggressive discipline and anxiety of preschoolers in China. Child Abuse & Neglect.

[B35-behavsci-15-01443] LeBuffe P., Naglieri J. (2013). Devereux early childhood assessment for preschoolers second edition (DECA-P2): User’s guide and technical manual.

[B36-behavsci-15-01443] Li J., Willems Y., Stok F., Dekovic M., Bartels M., Finkenauer C. (2019). Parenting and self-control across early to late adolescence: A three-level meta-analysis. Perspectives on Psychological Science.

[B37-behavsci-15-01443] Lin X., Xie W., Liao Y. (2024). The mediating role of negative parenting and children’s mastery motivation on socioeconomic Status and Chinese preschoolers’ problem behaviors. Early Education and Development.

[B38-behavsci-15-01443] Liu Z., Li J., Liang C. (2024). The impact of family socioeconomic status on students’ social and emotional skills: The serial mediation role of growth mindset and test and class anxiety. Journal of East China Normal University (Educational Sciences).

[B39-behavsci-15-01443] LoParo D., Fonseca A. C., Matos A. P. M., Craighead W. E. (2024). Anxiety and depression from childhood to young adulthood: Trajectories and risk factors. Child Psychiatry & Human Development.

[B40-behavsci-15-01443] Luo J., Van Grieken A., Kruizinga I., Raat H. (2023). Longitudinal associations between socioeconomic status and psychosocial problems in preschool children. European Child & Adolescent Psychiatry.

[B41-behavsci-15-01443] Mahoney J., Gucciardi D., Mallett C., Ntoumanis N. (2014). Adolescent performers’ perspectives on mental toughness and its development: The utility of the bioecological model. Sport Psychologist.

[B42-behavsci-15-01443] Masten A. S. (2001). Ordinary magic: Resilience processes in development. The American Psychologist.

[B43-behavsci-15-01443] Masten A. S., Coatsworth J. D. (1998). The development of competence in favorable and unfavorable environments. Lessons from research on successful children. The American Psychologist.

[B44-behavsci-15-01443] Masten A. S., Garmezy N., Tellegen A., Pellegrini D. S., Larkin K., Larsen A. (1988). Competence and stress in school children: The moderating effects of individual and family qualities. Journal of Child Psychology and Psychiatry, and Allied Disciplines.

[B45-behavsci-15-01443] Mian N. D., Godoy L., Briggs-Gowan M. J., Carter A. S. (2012). Patterns of anxiety symptoms in toddlers and preschool-age children: Evidence of early differentiation. Journal of Anxiety Disorders.

[B46-behavsci-15-01443] Morgan T., Yang S., Liu B., Cao Y. (2020). A comparison of psychological resilience and related factors in Chinese firstborn and only children. Asian Journal of Psychiatry.

[B47-behavsci-15-01443] Muhtadie L., Zhou Q., Eisenberg N., Wang Y. (2013). Predicting internalizing problems in Chinese children: The unique and interactive effects of parenting and child temperament. Development and Psychopathology.

[B48-behavsci-15-01443] Niditch L. A., Varela R. E. (2018). A longitudinal study of inhibited temperament, effortful control, gender, and anxiety in early childhood. Child & Youth Care Forum.

[B49-behavsci-15-01443] Noyes R. (2002). Anxiety and its disorders: The nature and treatment of anxiety and panic. Clinical Psychology Review.

[B50-behavsci-15-01443] Peng A., Qiu X., Ji S., Hu D., Dong B., Song T., Huang C., Chen L. (2022). The impact of childhood parental loss on risk for depression and anxiety in adulthood: A community-based study in Southwest China. Journal of Affective Disorders.

[B51-behavsci-15-01443] Peverill M., Dirks M. A., Narvaja T., Herts K. L., Comer J. S., McLaughlin K. A. (2021). Socioeconomic status and child psychopathology in the United States: A meta-analysis of population-based studies. Clinical Psychology Review.

[B52-behavsci-15-01443] Pinquart M. (2017). Associations of parenting dimensions and styles with externalizing problems of children and adolescents: An updated meta-analysis. Developmental Psychology.

[B53-behavsci-15-01443] Podsakoff P. M., MacKenzie S. B., Lee J.-Y., Podsakoff N. P. (2003). Common method biases in behavioral research: A critical review of the literature and recommended remedies. The Journal of Applied Psychology.

[B54-behavsci-15-01443] Pollard J., Reardon T., Williams C., Creswell C., Ford T., Gray A., Roberts N., Stallard P., Ukoumunne O. C., Violato M. (2023). The multifaceted consequences and economic costs of child anxiety problems: A systematic review and meta-analysis. JCPP Advances.

[B55-behavsci-15-01443] Poole J., Dobson K., Pusch D. (2017). Childhood adversity and adult depression: The protective role of psychological resilience. Child Abuse & Neglect.

[B56-behavsci-15-01443] Robinson C. C., Mandleco B., Olsen S. F., Hart C. H. (1995). Authoritative, authoritarian, and permissive parenting practices: Development of a new measure. Psychological Reports.

[B57-behavsci-15-01443] Roubinov D. S., Boyce W. T. (2017). Parenting and SES: Relative values or enduring principles?. Parenting.

[B58-behavsci-15-01443] Roubinov D. S., Hagan M. J., Boyce W. T., Adler N. E., Bush N. R. (2018). Family socioeconomic status, cortisol, and physical health in early childhood: The role of advantageous neighborhood characteristics. Psychosomatic Medicine.

[B59-behavsci-15-01443] Rutter M. (1999). Resilience concepts and findings: Implications for family therapy. Journal of Family Therapy.

[B60-behavsci-15-01443] Ryan R. M., Deci E. L. (2000). Self-determination theory and the facilitation of intrinsic motivation, social development, and well-being. The American Psychologist.

[B61-behavsci-15-01443] Sheerin C., Lind M., Brown E., Gardner C., Kendler K., Amstadter A. (2018). The impact of resilience and subsequent stressful life events on MDD and GAD. Depression And Anxiety.

[B62-behavsci-15-01443] Spence S. H., Rapee R., McDonald C., Ingram M. (2001). The structure of anxiety symptoms among preschoolers. Behaviour Research and Therapy.

[B63-behavsci-15-01443] Steinberg L., Lamborn S. D., Darling N., Mounts N. S., Dornbusch S. M. (1994). Over-time changes in adjustment and competence among adolescents from authoritative, authoritarian, indulgent, and neglectful families. Child Development.

[B64-behavsci-15-01443] Steinsbekk S., Ranum B., Wichstrøm L. (2021). Prevalence and course of anxiety disorders and symptoms from preschool to adolescence: A 6-wave community study. Journal of Child Psychology and Psychiatry.

[B65-behavsci-15-01443] Strickhouser J. E., Sutin A. R. (2020). Family and neighborhood socioeconomic status and temperament development from childhood to adolescence. Journal of Personality.

[B66-behavsci-15-01443] Tolan Ö., Ugur G. (2024). The relation between psychological resilience and parental attitudes in adolescents: A systematic review. Current Psychology.

[B67-behavsci-15-01443] Waanders C., Mendez J., Downer J. (2007). Parent characteristics, economic stress and neighborhood context as predictors of parent involvement in preschool children’s education. Journal of School Psychology.

[B68-behavsci-15-01443] Wang M., Zhao J., Bai W. (2009). Psychometric analysis of preschool anxiety scale in Chinese culture. Chinese Journal of Clinical Psychology.

[B69-behavsci-15-01443] Wang Y., Liu F., Li Y., Lin D. (2024). Supporting children and adolescents developing in adversity: A scoping review of resilience-promoting interventions from a socioecological perspective. Current Opinion In Behavioral Sciences.

[B70-behavsci-15-01443] Werner E. E. (1993). Risk, resilience, and recovery: Perspectives from the Kauai longitudinal study. Development and Psychopathology.

[B71-behavsci-15-01443] Whalen D. J., Sylvester C. M., Luby J. L. (2017). Depression and anxiety in preschoolers: A review of the past 7 years. Child and Adolescent Psychiatric Clinics of North America.

[B72-behavsci-15-01443] Williams K., Ciarrochi J., Heaven P. (2012). Inflexible parents, inflexible kids: A 6-year longitudinal study of parenting style and the development of psychological flexibility in adolescents. Journal of Youth and Adolescence.

[B73-behavsci-15-01443] Wright K., Reiser S., Delparte C. (2017). The relationship between childhood health anxiety, parent health anxiety, and associated constructs. Journal of Health Psychology.

[B74-behavsci-15-01443] Wright M. O., Masten A. S. (2005). Resilience processes in development: Fostering positive adaptation in the context of adversity. Handbook of resilience in children.

[B75-behavsci-15-01443] Wu P., Robinson C., Yang C., Hart C., Olsen S., Porter C., Jin S., Wo J., Wu X. (2002). Similarities and differences in mothers’ parenting of preschoolers in China and the United States. International Journal of Behavioral Development.

[B76-behavsci-15-01443] Xiang J., Wu J., Lian C., Lin X. (2025). The effects of home quarantine duration, parental emotional intelligence, and family socioeconomic status on children’s anxiety during the pandemic: A survey of 29,550 parents. Psychology Research and Behavior Management.

[B77-behavsci-15-01443] Xu J., Chen X., Liu S., Weng X., Zhang H., Yi Z., Gao M., Han Z. (2024). Parental autonomy support and psychological control and children’s biobehavioral functioning: Historical cohort differences in urban China. Child Development.

[B78-behavsci-15-01443] Xu X., Hanafi Z., Gao L. (2025). Sex differences in how are mothers’ SES related to late adolescents’ emotional stability in China: The mediating role of maternal parenting styles. BMC Psychology.

[B79-behavsci-15-01443] Zhang L., Cao H., Lin C., Ye P. (2022). Family socio-economic status and Chinese Preschoolers’ anxious symptoms during the COVID-19 pandemic: The roles of parental investment, parenting style, home quarantine length, and regional pandemic risk. Early Childhood Research Quarterly.

[B80-behavsci-15-01443] Zhu Y., Chen X., Zhao H., Chen M., Tian Y., Liu C., Han Z. R., Lin X., Qiu J., Xue G., Shu H., Qin S. (2019). Socioeconomic status disparities affect children’s anxiety and stress-sensitive cortisol awakening response through parental anxiety. Psychoneuroendocrinology.

